# Chrysin induces cell death and inhibits migration and invasion in squamous cervical carcinoma using a three-dimensional cell culture model

**DOI:** 10.1590/1414-431X2025e14692

**Published:** 2025-08-29

**Authors:** N.L. Mari, M.V.F. de Souza, L.E. de F. Meirelles, A.R.B. de A. Carvalho, C.S. Shinobu-Mesquita, M.L. Bruschi, M.E.L. Consolaro, V.R.S. da Silva

**Affiliations:** 1 Programa de Pós-Graduação em Biociências e Fisiopatologia, Universidade Estadual de Maringá, Maringá, PR, Brasil; 2 Departamento de Farmácia, Universidade Estadual de Maringá, Maringá, PR, Brasil

**Keywords:** Cervical cancer, SiHa cells, Chrysin, Spheroids, 3D cultures, Antineoplastic agent

## Abstract

Cervical cancer remains a leading cause of cancer-related mortality among women worldwide, despite treatment advances. The most common form is squamous cell cervical carcinoma, primarily associated with human papillomavirus (HPV) type 16. Chrysin (5,7-dihydroxyflavone) is a natural flavonoid with promising anticancer properties both *in vitro* and *in vivo*. The aim of this study was to evaluate the antiproliferative, anti-migratory, and anti-invasive effects of chrysin on the SiHa human cervical cancer cell line (HPV-16-positive) using a 3D cell culture model with spheroids. Cell viability was assessed using the resazurin assay, while cytostatic effects were monitored by measuring spheroid size through imaging. Migration was evaluated with the spheroid migration assay. The expression of matrix metalloproteinase (MMP)-2, MMP-9, and vascular endothelial growth factor (VEGF) was quantified by immunoenzymatic assays. Chrysin treatment exhibited concentration-dependent cytotoxic and cytostatic effects, reducing cell proliferation and decreasing SiHa spheroid size. Additionally, chrysin inhibited cell migration and invasion, potentially reducing metastatic potential, primarily by decreasing the production of MMP-2 and VEGF. These findings suggest that chrysin has therapeutic potential for squamous cell cervical carcinoma and warrants further *in vivo* preclinical studies.

## Introduction

In 2020, over 606,000 women were diagnosed with cervical cancer, resulting in 341,831 deaths worldwide. Cervical cancer is the fourth most common cancer in women, accounting for 6.5% of all cancer cases (1). The primary cause of cervical cancer is persistent infection with human papillomavirus (HPV), particularly high-risk oncogenic types (2). Despite being a preventable disease due to the availability of effective screening methods (e.g., cytology and HPV testing) and preventive vaccines (3), cervical cancer remains one of the most prevalent cancers and a leading cause of cancer-related deaths among women globally (1).

**Figure 1:**
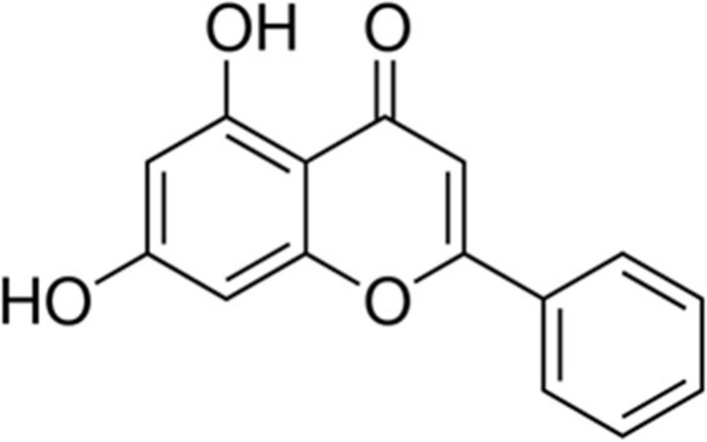
Chemical structure of chrysin (5).

Although significant therapeutic advances have been made in recent years, current cancer therapies remain insufficient, as many treatments cause side effects that compromise the patient's quality of life, and drug resistance can limit treatment efficacy (4). Chrysin (5,7-dihydroxyflavone) (Figure 1) is a natural flavonoid found in honey, propolis, and various plants, primarily those of the *Scutellaria* genus (5,6). Chrysin has shown promising antitumor properties in both *in vivo* tumor models and *in vitro* human cancer cell lines, including leukemia, breast, prostate, ovarian, glioma, hepatocellular, and esophageal cancers (7-11). However, there is limited research on chrysin in cervical cancer, with most studies focusing on the HeLa cell line, which contains integrated HPV-18 (12-15). To date, few studies have explored the inhibitory effects of chrysin on HPV-16-positive cell lines (such as SiHa), the most prevalent HPV type and the leading cause of squamous cell cervical carcinoma (1). Squamous cell cervical carcinoma is the most common type of cervical cancer worldwide, accounting for approximately 90% of cases (1,2).

To the best of our knowledge, no studies have evaluated the cytotoxic effects of chrysin in a three-dimensional (3D) cell culture model for cervical cancer. 3D cell culture systems, in contrast to traditional 2D monolayers, better mimic the tumor microenvironment, offering more accurate representations of the malignant phenotype, including factors like cell-cell interactions, drug metabolism, and sensitivity (16,17). The spheroid model, commonly used in 3D culture systems, provides sufficient complexity to replicate aspects of human tissues and tumors (18).

In this study, we investigated the antiproliferative, anti-migratory, and anti-invasive effects of chrysin on the SiHa cervical cancer cell line, which is HPV-16-positive and derived from human squamous cell carcinoma, using a 3D spheroid model.

## Material and Methods

### Reagents and cell line

Chrysin (5,7-dihydroxyflavone), Dulbecco′s Modified Eagle′s Medium (DMEM), amphotericin B, dimethylsulfoxide (DMSO), and sodium resazurin salt (7-hydroxy-3H-phenoxazin-3-one-10-oxide sodium salt) were purchased from Sigma Aldrich (USA). Fetal bovine serum (FBS), penicillin/streptomycin, agarose, enzyme-linked immunosorbent kit (ELISA) for human MMP-2, MMP-9, and vascular endothelial growth factor (VEGF) were purchased from Invitrogen (USA).

The SiHa cell line, derived from human invasive squamous cell cervical carcinoma (containing 1 to 2 copies of integrated HPV-16 per cell; HTB-35, ATCC), was generously provided by Dr. Luisa L. Villa, Faculty of Medicine, University of São Paulo, SP, Brazil, and Dr. Silvya S. Maria-Engler, Faculty of Pharmaceutical Sciences, University of São Paulo. Cells were cultured in DMEM supplemented with 10% FBS, 100 U/mL penicillin, 100 μg/mL streptomycin, and 2.5 μg/mL amphotericin B, and maintained at 37°C in a humidified incubator with 5% CO_2_.

### SiHa spheroid generation

SiHa spheroids were generated using a modified method based on cell culture microplates with U-bottom wells coated with agar (19). Briefly, 96-well U-bottom culture plates were coated with 80 μL of 1.5% (v/v) agarose to prevent cell adhesion. After agarose polymerization, SiHa cells (between passages 3 and 8) were seeded at a density of 2×10^4^ cells per well. The plates were incubated at 37°C in a humidified atmosphere with 5% CO_2_ for six days. Every two days, 100 μL of DMEM medium was replaced in each well. Spheroid formation was monitored using an inverted microscope (EVOS FL Cell Imaging System, Life technologies, USA) at 24, 48, 72, and 96 h. Images were captured at 96-h post-seeding. All subsequent assays were performed 96 h after spheroid formation.

### Cytotoxicity assay

Chrysin (Sigma-Aldrich) was dissolved in DMSO at a concentration of 40 mM and stored at -20°C. SiHa spheroids were exposed to chrysin at concentrations ranging from 25 to 1600 μM (1/2 dilution) for 72 h, based on previous studies in monolayer cultures (12), with adjustments for higher concentrations in the 3D model. DMSO-treated spheroids served as negative controls (NC). Cell viability was assessed using the resazurin reduction assay (20). After 48 h of treatment, 10 μL of 5 nM resazurin solution was added to each well, followed by an additional 24-h incubation in the CO_2_ incubator. Absorbance was measured at λexc=535/40 and λemis=600/40, and cell viability was calculated.

The concentration-response curve was generated, and the IC_30_, IC_50_, and IC_90_ inhibitory concentrations (concentrations inhibiting cell growth by 30, 50, and 90%, respectively, compared to NC) were determined by non-linear regression analysis using GraphPad Prism 8.0 (GraphPad Software, USA).

### Cytostatic assay

For the cytostatic assay, SiHa spheroids were treated with chrysin (IC_50_) for 72 h. Spheroids treated with DMEM alone served as NC. Spheroid growth after 72 h was assessed using an inverted microscope, with images captured every 24 h. Spheroid volume (V) was calculated by measuring the two crossed diameters and applying the formula (V=a.b^2^·π/6), where “a” and “b” represented the smaller and larger diameters, respectively (20).

### Cell migration and invasion assays

After treatment with chrysin (IC_50_), cell migration was monitored every 24 h using an inverted microscope and after 72 h, images were taken under 100× and 200× magnification (EVOS FL Cell Imaging System, Life Technologies, USA). Images were analyzed using Motic Image Plus 3.0^©^ software (Motic, China). Total spheroid diameter was measured at each time point, and the distance traveled by the cells was calculated by subtracting the diameter at 24, 48, and 72 h from the initial diameter (21).

For invasion assays, SiHa spheroids were treated with chrysin (IC_50_), with DMEM-treated spheroids serving as NC. After 72 h of treatment, supernatants were collected, centrifuged (15 *g*, 1 min, 4°C), and analyzed according to the manufacturer's instructions if the Elisa kits (Invitrogen, USA). Absorbance was measured at 450 nm using a microplate reader (Loccus, Brazil). The levels of MMP-2, MMP-9, and VEGF were quantified using ELISA kits (Human MMP-2 ELISA kit, Human MMP-9 ELISA kit, and Human VEGF-B ELISA kit, Invitrogen, USA).

### Statistical analysis

Data were analyzed using analysis of variance (ANOVA) followed by the Tukey-Kramer multiple comparisons test, except for viability experiments, where a *t*-test was used. At least three independent experiments were performed, and results are reported as means±SD. GraphPad Prism 8.0 software (GraphPad) was used for statistical analysis. P-values <0.05 were considered statistically significant.

## Results and Discussion

The present study aimed to evaluate the efficacy of chrysin against the SiHa cervical cancer cell line using a 3D cell culture model with spheroids.

**Figure 2:**
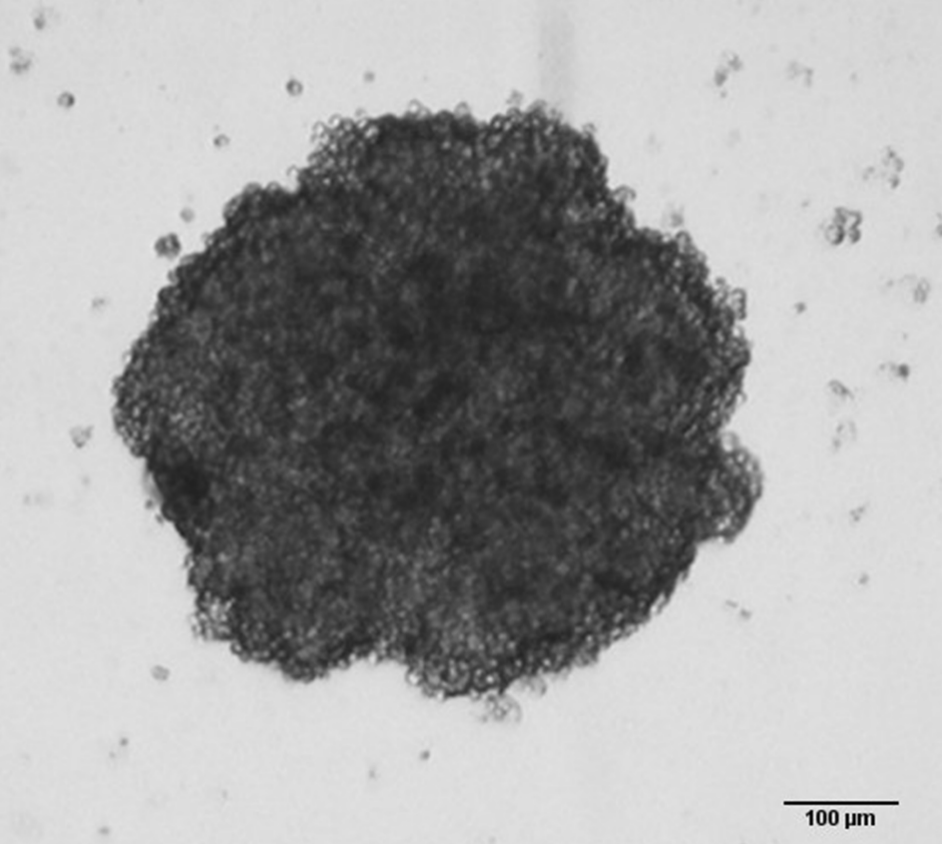
SiHa spheroids generated using the liquid overlay method after 96 h. Optical microscopy images (100× magnification). Scale bar=100 µm.

SiHa spheroids were generated using the liquid overlay method. In this technique, 96-well U-bottom plates were coated with agarose to prevent cell adhesion, allowing cells to aggregate and form a single spheroid per well (22). This method enables the simultaneous generation of a large number of spheroids on a single plate, improving experimental reproducibility and facilitating monitoring of spheroid formation and growth (16). Figure 2 shows that the technique used was effective in generating the SiHa spheroids after 96h.

### Chrysin exhibited cytotoxic and cytostatic effects on SiHa spheroids

**Figure 3:**
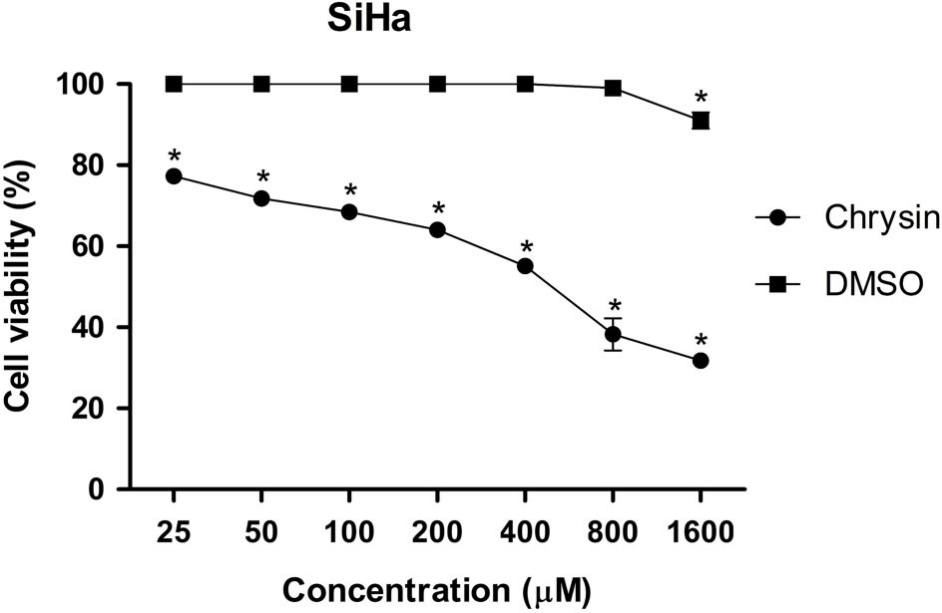
Concentration-response curve indicating the viability of spheroid cells of the cervical cancer cell line (SiHa) after exposure to different concentrations of chrysin (25-1600 μM) for 72 h. The DMSO control maintained 100% viability at almost all concentrations. Data are reported as means±SD of three separate experiments conducted in triplicate. *P<0.005, compared to the negative control (*t*-test).

To analyze the effects of chrysin on the SiHa cervical cancer cell line in a 3D culture system, SiHa spheroids were exposed to increasing concentrations of chrysin (25 to 1600 μM) for 72 h. Chrysin treatment reduced cell viability and exhibited concentration-dependent cytotoxic effects on SiHa spheroids, with significant decreases in viability observed from the lowest concentration tested (P<0.0001) (Figure 3). Spheroids treated with DMSO alone showed a reduction in viability only at the highest concentration, suggesting that the cytotoxic effects are specific to chrysin. The IC_30_, IC_50_, and IC_90_ values for chrysin on SiHa spheroids were 292.3, 487.2, and 876.9 μM, respectively. The IC_50_ value indicated a concentration-dependent cytotoxic effect.

To our knowledge, this is the first study to evaluate the effects of chrysin on 3D spheroid models derived from a cervical cancer cell line. Previous studies in monolayer cultures have reported IC_50_ values for HeLa cells ranging from 4.6 to 31.25 μM (13-15,23-25) and for CaSki cells from 5.1to 17.8 μM (14,25), which are significantly lower than the IC_50_ value observed for SiHa spheroids in this study. This difference may be attributed to the fact that 2D monolayer cultures do not replicate the complex multicellular organization found *in vivo* (16). In contrast, 3D spheroid models more accurately represent human tumors and are better suited for drug screening (17). Therefore, the IC_50_ value for chrysin in SiHa spheroids is likely more reflective of *in vivo* conditions.

These findings are consistent with other studies demonstrating cytotoxic effects of chrysin in various cancer models, including HeLa (13-15,23-25) and CaSki cells (14,25), and in spheroid models of colon cancer (SW620) (26) and osteosarcoma (MG63) (27).

However, considering the relatively high IC_50_ observed in this study, strategies to improve bioavailability of chrysin could be explored in the future to enhance its potential, reducing the dose required for an effective cytotoxic effect. In this regard, other studies using breast cancer cells or human ovarian carcinoma cells were conducted incorporating chrysin into nanoparticles (28,29) or using combined therapy (30). Additionally, chrysin has been shown to enhance the cytotoxicity of doxorubicin and cisplatin in squamous cell carcinoma spheroids of the human lung (RERF-LC-AI) (31).

**Figure 4:**
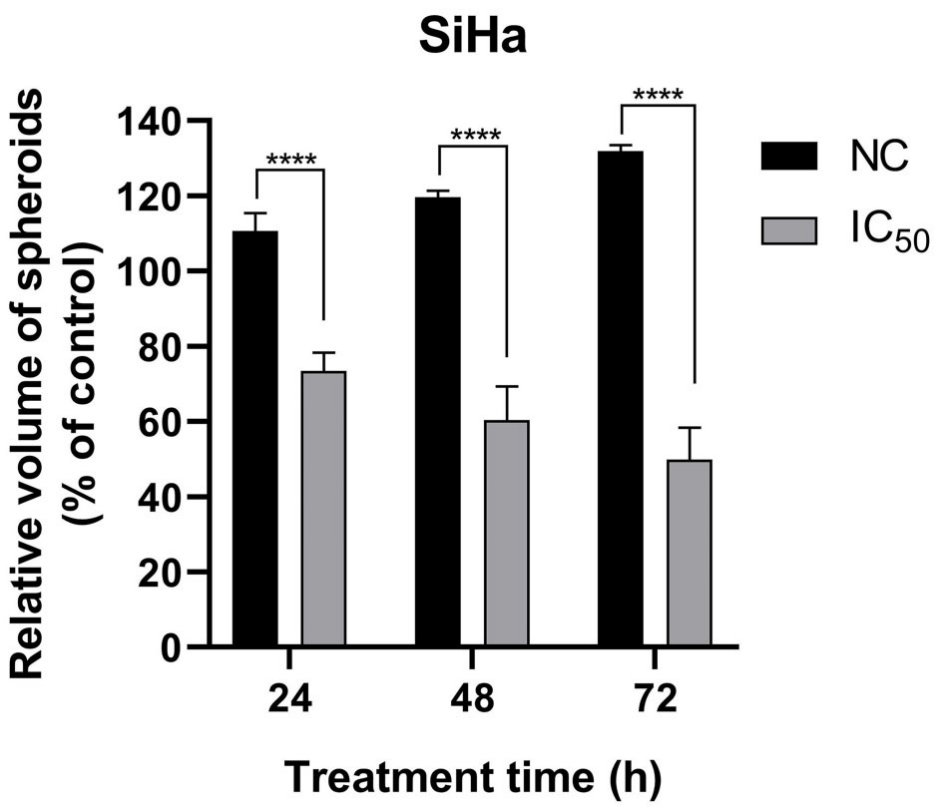
Effects of chrysin treatment (concentration inhibiting 50% cell growth (IC_50_= 487.2 μM)/72 h) on SiHa spheroid growth by the cytostatic test. The graph represents the variation in the volume of the spheroid in relation to the negative control (NC) after treatment. Data are reported as means±SD of three separate experiments conducted in triplicate. ****P<0.0001 at 24, 48, and 72 h of treatment compared to the NC (ANOVA followed by the Tukey-Kramer multiple comparisons test).

To further assess the impact of chrysin on spheroid growth, we conducted a cytostatic assay. SiHa spheroids treated with chrysin (IC_50_) for 72 h exhibited a statistically significant reduction in volume (P<0.0001) compared to the NC spheroids at the respective time points (Figure 4). Consistent with the cytotoxicity results, chrysin treatment led to a marked inhibition of SiHa spheroid growth. These findings align with a previous study demonstrating a cytostatic effect of chrysin on colorectal cancer cell lines (SW480 and HCT-116) in monolayer cultures (32).

### Chrysin inhibited cell migration and invasion in SiHa spheroids

**Figure 5:**
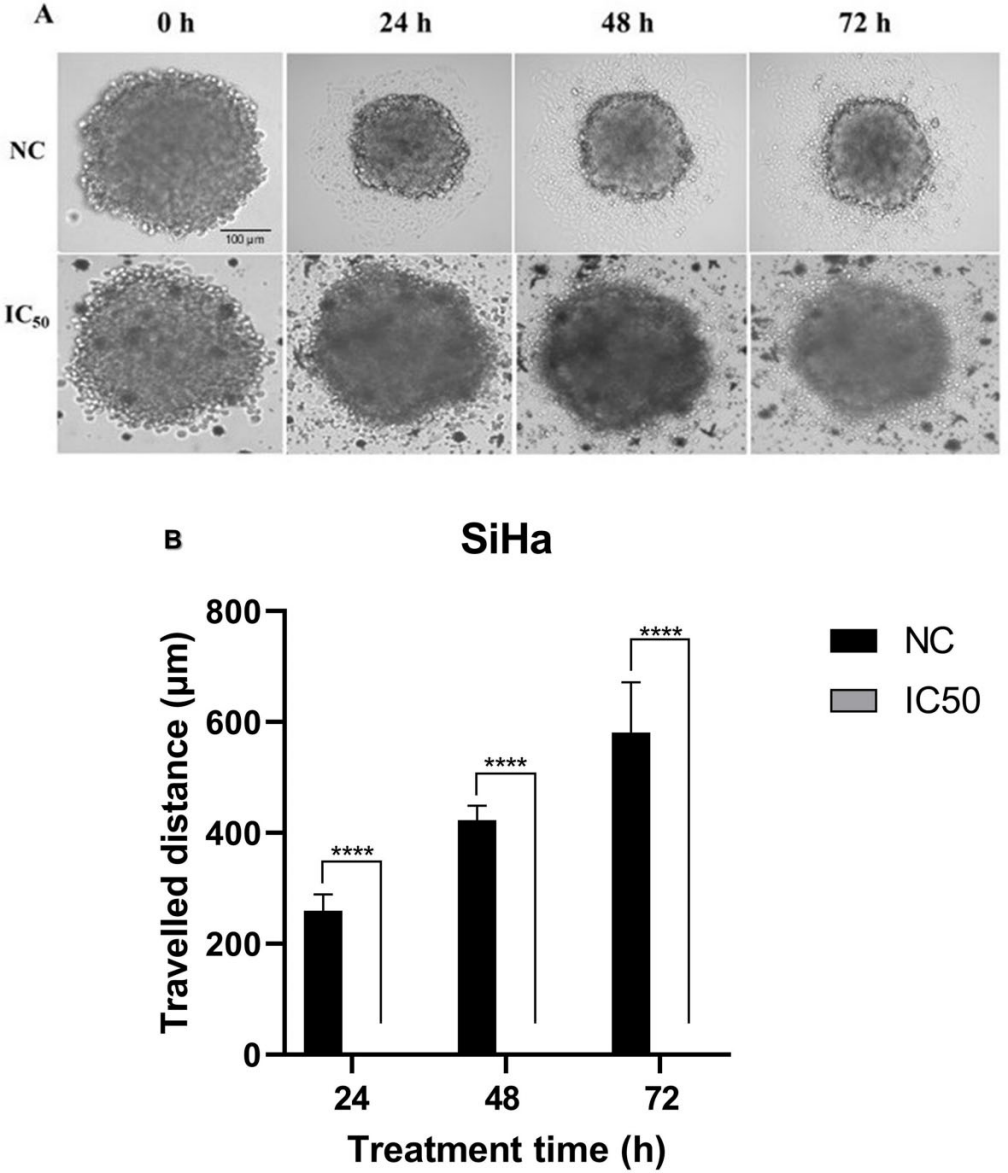
. Effects of chrysin treatment (concentration inhibiting 50% cell growth (IC_50_= 487.2 μM)/72 h) on SiHa spheroid cell migration. **A**, Spheroid optical microscopy images (100× magnification; scale bar=100 µm). In the negative control (NC) spheroids, peripheral cell migration was observed up to 72 h of incubation, whereas migration was minimal in the spheroids treated with chrysin up to the same incubation time. **B**, Distance traveled by the cells from the spheroids. Data are reported as means±SD of three separate experiments conducted in triplicate. ****P<0.0001 (ANOVA followed by the Tukey-Kramer multiple comparisons test).

The spheroid migration assay demonstrated that SiHa spheroids treated with chrysin (IC_50_) exhibited minimal cell migration over a 72-h period, in contrast to NC spheroids, where peripheral cells migrated up to 72 h (Figure 5A and B). This difference in migration was statistically significant (P<0.0001) at 24, 48, and 72 h, suggesting that chrysin effectively inhibits cell migration in SiHa spheroids. This result is consistent with studies showing similar effects of chrysin on migration in other cancer models, including colorectal cancer (SW480 and HCT-116 cells) (32) and melanoma (A375 cells) (33).

Cell invasion, a critical process in metastasis, was also assessed. Tumor cells invade the extracellular matrix and basement membrane, potentially leading to metastasis, which worsens prognosis and complicates treatment (34). MMPs, specifically MMP-2 and MMP-9, play key roles in extracellular matrix degradation and tumor invasion (35). VEGF is involved in angiogenesis, supporting tumor growth and metastasis (36).

**Figure 6:**
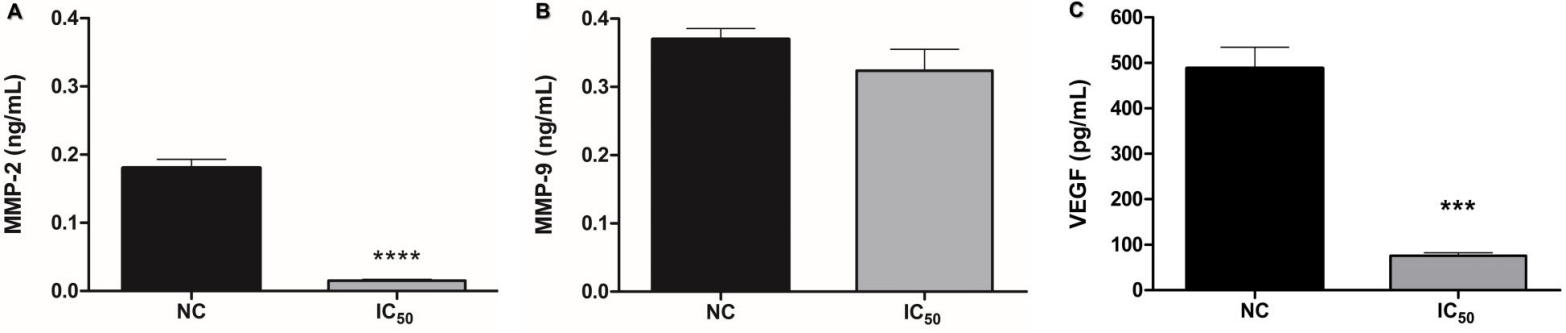
Effects of chrysin treatment (concentration inhibiting 50% cell growth (IC_50_= 487.2 μM/72 h) on SiHa spheroid cell invasion parameters at 72 h of treatment including the levels of matrix metalloproteinase (MMP)-2 (**A**), MMP-9 (**B**), and vascular endothelial growth factor (VEGF) (**C**) quantified using enzyme-linked immunosorbent (ELISA) assays. Data are reported as means±SD of three separate experiments conducted in triplicate. ****P<0.0001, ***P=0.0009 (ANOVA followed by the Tukey-Kramer multiple comparisons test).

To evaluate the effects of chrysin on invasion, we measured MMP-2, MMP-9, and VEGF levels in the supernatants of SiHa spheroids after 72 h of chrysin treatment using ELISA. A significant decrease was observed in MMP-2 (P<0.0001) and VEGF (P=0.0009) levels (Figure 6A and C), but no significant change was observed in MMP-9 levels (P=0.1864) (Figure 6B). While the reduction in MMP-2 and VEGF levels is consistent with previous studies showing chrysin's effects in other cancer models (37-39), the lack of change in MMP-9 levels in SiHa spheroids warrants further investigation. However, these results can be explained by previous studies that show that MMP-2 and MMP-9 may exhibit different roles in cervical tumor progression, where MMP-2 can be associated with aggressive behavior, while MMP-9 expression can be associated with a favorable prognosis (40).

Taken together, these data suggest that chrysin not only exerts cytotoxic and cytostatic effects on SiHa spheroids but also may reduce the migration and invasive potential of squamous cervical carcinoma cells by inhibiting cell migration and invasion through the suppression of MMP-2 and VEGF. These data are consistent with the literature, which shows that chrysin possesses antiproliferative properties, potentially inhibiting metastasis and invasion (7,8,13-15,23).

The present study is extremely important as it evaluated the chrysin effect in an HPV-16-positive cell line using a 3D culture model that provides sufficient complexity to replicate aspects of human tissues and tumors. However, it has limitations such as the use of an *in vitro* model with only one type of cervical cancer cell, which may restrict the generalization of the results to other types of cervical cancer cells. Therefore, further studies that explore different cell types and evaluation of *in vivo* models could validate the indication of chrysin for treating cervical cancer and expand the findings presented in this work.

## Conclusions

Our findings demonstrated that chrysin exerted concentration-dependent cytotoxic and cytostatic effects on SiHa spheroids, leading to a reduction in cell proliferation and a decrease in spheroid size. Furthermore, chrysin treatment effectively inhibited cell migration and invasion, likely by downregulating the production of MMP-2 and VEGF. These results highlight the potential of chrysin as a promising therapeutic candidate for squamous cell cervical carcinoma and underscore the need for further *in vivo* preclinical studies to assess its efficacy.
